# Died under Gas

**Published:** 1896-02

**Authors:** 


ARTICLE VII.

### DIED UNDER GAS.

Mrs. Flora Josephs, of No. 418 East Eighty-second street, died a short time since in the office of Dr. Robert Wolf of No. 315 East Eighty sixth street, New York, while under the influence of nitrous oxide gas.

Mrs. Josephs, who was twenty-two years old, was the mother of a four year old boy. Her husband has for some years been employed as a salesman in a grocery store, but has been out of work for the last two months.

Mrs. Josephs went to Dr. Wolf's office about 3.30 o'clock yesterday. She told the dentist that she had been suffering for a long time from four badly decayed teeth, and wanted them extracted. She neglected to give Dr. Wolf her name, simply saying that Mrs. Steinhart, a patient of his, had sent her to him.

She was afraid of the pain of having the teeth extracted, asked Dr. Wolf to give her gas.

"I asked her if she had ever taken gas," said Dr. Wolf last night, "and she told me she had not. I questioned her closely about the state of her health, and made the usual examination to ascertain whether she could stand the anæsthetic. She appeared to be healthy in every way.

"Just before I administered the gas she said she would like to have her husband present, and I told her to go or send for him if she wished. She talked about it for a moment, and finally decided not to send for him.

"I administered the gas carefully, and noticed that she seemed to take it very easily. She fell to sleep very peacefully and kept quite still while I extracted the teeth. Just as I was finishing the operation, I noticed that she stopped breathing. I rang for assistance, and several ladies who live in the house came in. One of them called Dr. Churchill, whose office is next door.

"In the meantime I had given the patient repeated hypodermic injections of whiskey, and lowered the back of the operating chair so that the blood would go to her head. When Dr. Churchill came in we tried artificial respiration, but with no result. Finally I gave her an injection of cocaine, and later more injections of whiskey. We worked hard for an hour to revive her, but it was no use."

When asked what he thought the cause of death was, Dr. Wolf said he believed Mrs. Josephs had some organic disease of the heart. The gas used was the same kind, he said, that he had used for four years.

Dr. Wolf did not know the dead woman's name, and after notifying a policeman, he went to the home of Mrs. Steinhart; of No. 146 East Eighty-ninth street. He was told that Mrs. Steinhart would not be home until 7 o'clock. He then remembered that Mrs. Steinhart had a cousin named Charles Josephs, who lived at No. 418 East Eighty-second street.

No one was in the Joseph's flat when he rang the bell.

Mrs. Simmons, who lived across the hall, came out and told him that Mrs. Josephs had gone to Dr. Wolf's office in East Eighty-sixth street. She was taking care of Mrs. Joseph's child. This convinced Dr. Wolf that the dead woman was Mrs. Josephs. She was afterwards identified by Mrs. Steinhart and Mrs. Simmons.

Coroner O'Meagher, who had been notified by the police, reached Dr. Wolf's office about 3.40 o'clock. After examining the body and questioning Drs. Wolf and Churchill, he gave a permit for its removl. The coroner exonerated Dr. Wolf from all blame, saying that the woman's death was purely an accident.

Dr. Wolf went to Joseph's house and told him of his wife's death. In the conversation which followed it was learned that Joseph, who is thirty years old, had been befriended by Dr. Wolf's father when he first came to this country from Germany.—N. Y. World.

(This is only another case in which the asphyxia produced by the gas was carried beyond the point or line for recovery. Whenever the affinity of the blood has been so weakened by deoxidation that it fails to take up the oxygen from the atmosphere, then the patient sinks, and all the powers of earth cannot restore to life.

This woman was in good health, but what of that if deprived of the force of life; which is free oxygen, as is always the case in getting absensation by the use of nitrous oxide gas. Therefore, as Dr. Richardson says, "its use should be prohibited by law."—Editor.)—*D. & S. Microcosm.*

